# Amick vs. Reeves

**Published:** 1895-08

**Authors:** 


### AMICK *VS.* REEVES.

We have several times alluded in these pages to the damage suit pending in the United States court at Chattanooga under the above title. The suit was tried at the April term and a verdict very properly rendered for the defendant.

Dr. Reeves has lately had printed a pamphlet containing a great part of the court proceedings, the allegations, arguments of counsel, the judge's charge, etc., in which the methods of quack Amick and his "chemical company" are fully exposed to the light. Dr. Reeves made a brave and successful fight against one of the boldest and most bare-faced frauds that was ever offered to an intelligent people, and he richly deserves the gratitude of the entire profession. If one wishes to learn of the ingenious but shameless tricks to which the Amick crowd resorted in order to foist their so-called cure upon both the public and the medical profession, he has but to read the evidence produced at this trial by Dr. Reeves. Amick, himself once a physician in good standing among his Cincinnati confrères, has sold his professional birthright for a pottage of stock in a secret medicine humbug, thereby placing himself outside the limits of respectable professional relationship.

The pamphlet alluded to makes very interesting reading. It may be had by addressing Dr. James E. Reeves, Chattanooga, Tennessee.

